# Dr. Boiling’s Address at Louisville

**Published:** 1875-06

**Authors:** 


The President of the American Medical Association concluded his address as follows:

Gentlemen, Western Medicine, after a long time, established its Mecca at the Falls of the Ohio. Whatever the fashioners of taste may determine, the medical heart cannot go far astray in recalling the Titans that officiated at its altars. Many of them "sleep well after life's fitful fever," but the rock-girt and rock-floored river in the neighborhood of their ashes, as it throws its disturbed waters over the cascade, will chant their requiem while grass grows or water runs. One, in a green old age, whose fame has filled the world, stands, like the statue of a demigod, poised on the apex of his monumental shaft, far above all surrounding things, pointing to an earlier day-star than greets the vision of ordinary mortality. Another, happy in the memories of a well-spent life, the charming grace of whose cultured pen has left an imperishable record, lingers in the peaceful enjoyment of that subdued and enchanting twilight of life between sundown and the "deeper gloaming," so in harmony with the spirit of the good, having thrown his mantle on other shoulders, patiently awaits the translation. One, the Galen now of the great city of the Republic, garners the golden sheaves of a crop sown long ago, and thoroughly cultivated. Another, the American Dupuytren, on the fringe of the sunny land of the orange and the magnolia, with the premonitions of a glorious sunset gathering about him, in faith and hope is also ready. We know that their example is not lost on those who have taken their places in the flourishing medical institutions of this noble city—a city whose munificence to medicine has entitled it forever to the kindest memories of the profession.—*Clinic.*

